# Early and late endothelial response in breast cancer metastasis in mice: simultaneous quantification of endothelial biomarkers using a mass spectrometry-based method

**DOI:** 10.1242/dmm.036269

**Published:** 2019-03-01

**Authors:** Joanna Suraj, Anna Kurpińska, Agnieszka Zakrzewska, Magdalena Sternak, Marta Stojak, Agnieszka Jasztal, Maria Walczak, Stefan Chlopicki

**Affiliations:** 1 Jagiellonian University, Jagiellonian Centre for Experimental Therapeutics, Bobrzynskiego 14, 30–348 Krakow, Poland; 2 Jagiellonian University Medical College, Faculty of Pharmacy, Chair and Department of Toxicology, Medyczna 9, 30–688 Krakow, Poland; 3 Jagiellonian University Medical College, Faculty of Medicine, Chair of Pharmacology, Grzegorzecka 16, 31–531 Krakow, Poland

**Keywords:** Endothelium, Murine 4T1 model of breast cancer, Metastasis, Biomarkers, MicroLC/MS-MRM

## Abstract

The endothelium plays an important role in cancer metastasis, but the mechanisms involved are still not clear. In the present work, we characterised the changes in endothelial function at early and late stages of breast cancer progression in an orthotopic model of murine mammary carcinoma (4T1 cells). Endothelial function was analysed based on simultaneous microflow liquid chromatography–tandem mass spectrometry using multiple reaction monitoring (microLC/MS-MRM) quantification of 12 endothelium-related biomarkers, including those reflecting glycocalyx disruption – syndecan-1 (SDC-1), endocan (ESM-1); endothelial inflammation – vascular cell adhesion molecule 1 (VCAM-1), intercellular adhesion molecule 1 (ICAM-1), E-selectin (E-sel); endothelial permeability – fms-like tyrosine kinase 1 (FLT-1), angiopoietin 2 (Angpt-2); and haemostasis – von Willebrand factor (vWF), tissue plasminogen activator (t-PA), plasminogen activator inhibitor 1 (PAI-1), as well as those that are pathophysiologically linked to endothelial function – adrenomedullin (ADM) and adiponectin (ADN). The early phase of metastasis in mouse plasma was associated with glycocalyx disruption (increased SDC-1 and ESM-1), endothelial inflammation [increased soluble VCAM-1 (sVCAM-1)] and increased vascular permeability (Angpt-2). During the late phase of metastasis, additional alterations in haemostasis (increased PAI-1 and vWF), as well as a rise in ADM and substantial fall in ADN concentration, were observed. In conclusion, in a murine model of breast cancer metastasis, we identified glycocalyx disruption, endothelial inflammation and increased endothelial permeability as important events in early metastasis, while the late phase of metastasis was additionally characterised by alterations in haemostasis.

## Abstract (precis)

**Summary:** A microLC/MS-MRM-based approach for simultaneous determination of endothelium-related biomarkers identified glycocalyx disruption, endothelial inflammation and increased endothelial permeability as important events in early pulmonary metastasis in a murine model of breast cancer metastasis.

## INTRODUCTION

Despite the considerable progress in diagnostics and treatment of cancer, mechanisms of metastasis are still not fully understood and constitute an important barrier for the development of effective anti-metastatic therapy (Zheng et al., 2013). Indeed, treatment failure presently occurs in approximately 30% of breast cancer patients (Mullan and Millikan, 2007). An important limitation in current treatment strategies is the lack of reliable plasma biomarkers that would allow for early detection of tumour metastasis and monitoring of therapy efficacy. Recent attention has been paid to the analysis of multi-biomarkers instead of a single-molecule approach as the simultaneous imaging of multiple processes potentially involved in metastasis may enable better identification of disease progression and mechanisms underlying formation of metastases. Such a multi-biomarker approach could be effectively used not only in early diagnosis, but also in the selection of preventive and therapeutic methods, potentially contributing to the development of precision oncology (Mannello and Ligi, 2013; Schiffman et al., 2015; Gallo Cantafio et al., 2018; Signoretti et al., 2018).

In recent years, attention has been focused on the significance of the vascular endothelium in cancer metastasis. Healthy endothelial cells inhibit tumour cell adhesion, transmigration and metastasis formation (Blazejczyk et al., 2015), whereas endothelial dysfunction, characterised by a shift towards decreased synthesis of vasoprotective mediators and activation of pro-inflammatory and pro-thrombotic molecules (Walczak et al., 2015; Frołow et al., 2015), promotes cancer metastasis (Blazejczyk et al., 2015). In fact, tumour cells respond to the chemokines and cell adhesion molecules engaged in leukocyte trafficking (Roblek et al., 2015; Schlesinger and Bendas, 2015). Abundant evidence suggests that endothelial inflammation enhances the adhesion of cancer cells to the endothelium and plays a major part in the development of metastases (Franses et al., 2013; Rajendran et al., 2013). By contrast, vasoprotective mediators, such as nitric oxide (NO) and carbon monoxide (CO), inhibit cancer cell adhesion and transmigration through the endothelium (Vahora et al., 2016; Kourti et al., 2017; Stojak et al., 2018), while prostacyclin (PGI_2_) and PGI_2_-releasing compounds have anti-metastatic effects (Minami et al., 2015; Blazejczyk et al., 2016).

Given the key role of the endothelium in metastasis formation, the aim of this study was to characterise changes in endothelial function that take place during the early and late phases of breast cancer development in mice using a microflow liquid chromatography–tandem mass spectrometry using multiple reaction monitoring (microLC/MS-MRM)-based method that enables simultaneous determination of specific sequences characteristic for 11 proteins and one peptide biomarker defining endothelial function (Suraj et al., 2018, 2019).

The panel of selected biomarkers included proteins involved in various processes linked to endothelial dysfunction: glycocalyx disruption – syndecan-1 (SDC-1), endocan (ESM-1); endothelial inflammation – vascular cell adhesion molecule 1 (VCAM-1), intercellular adhesion molecule 1 (ICAM-1), E-selectin (E-sel; also known as Sele); vascular permeability – fms-like tyrosine kinase 1 (FLT-1), angiopoietin 2 (Angpt-2); and haemostasis – von Willebrand factor (vWF), tissue plasminogen activator (t-PA; also known as Plat), plasminogen activator inhibitor 1 (PAI-1; also known as Serpine1), as well as other proteins/peptides related to endothelial function – adrenomedullin (ADM), adiponectin (ADN; also known as Acrp30 and Adipoq). The selected proteins represent secreted proteins, receptors or cleaved surface proteins circulating within plasma (Paulus et al., 2011; Walczak et al., 2015). In order to study the progressive endothelial response to primary tumour growth and breast cancer metastasis, we took advantage of the murine model of spontaneously metastasising 4T1 mammary gland carcinoma with prolonged development of metastasis (Pulaski and Ostrand-Rosenberg, 2001; Buczek et al., 2018; Bailey-Downs et al., 2014). This approach permitted quantification of biomarker concentration in plasma in relation to primary tumour growth in early and late phases of lung metastasis. Furthermore, we evaluated the time course of changes of biomarkers not only in plasma, but also in lungs and primary tumours, allowing determination of a possible cellular origin of proteins/peptides circulating in plasma.

## RESULTS

### Basic characteristics of primary tumour and development of metastasis in the lungs

**Fig. 1.**
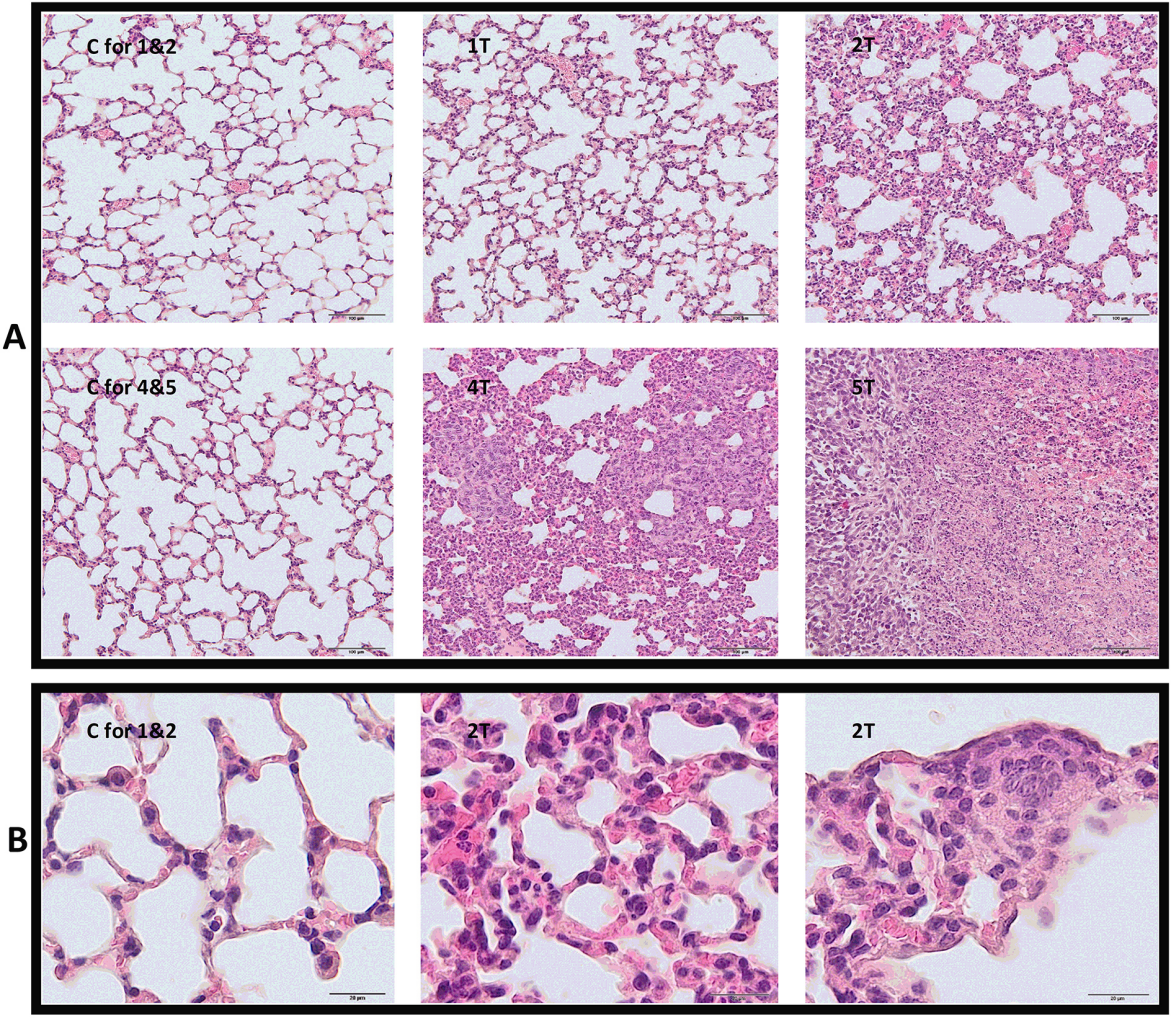
**Histopathological analysis of the Haematoxylin- and Eosin-stained lungs.** (A) Histology of the lungs in subsequent weeks after 4T1 tumour cell inoculation. (B) Higher-magnification microphotographs confirming the presence of inflammation, hyperaemia, reduction in alveolar volume and micrometastases 2 weeks after tumour cell inoculation in comparison to control group. C, control group (respective control groups are presented: C for 1&2 and C for 4&5); 1T, 2T, 4T, 5T, groups of tumour-bearing mice in subsequent (1, 2, 4 and 5) weeks after tumour cell inoculation. Scale bars: 100 µm (A), 20 µm (B).

Primary tumours (0.13±0.07 g) were detectable in the second week after cancer cell inoculation, and the mean tumour weight progressively increased throughout the course of the study to 2.39±1.13 g in the fifth week, while tumour volume rose from 95 cm^3^ in the second week to 1195 cm^3^ in the fifth week after 4T1 cell inoculation. The onset of metastatic foci formation was macroscopically observed in the lungs beginning from the second week, but only a single metastatic nodule in one mouse per six was noted, and increasing thereafter, finally resulting in 33 metastatic foci in the fifth week (Table 1). Histopathological analysis of the lungs revealed early (first week) and progressing development of metastasis-related inflammation. The onset of the micrometastases formation was noted in the second week after cancer cell inoculation (Fig. 1). The robust metastasis development was noted in the fourth and fifth week after tumour cell inoculation. Based on these results, the first 2 weeks after tumour cell inoculation were defined as an early metastatic period and the fourth and fifth weeks as the late metastatic phase – compatible with our previous work with this model (Buczek et al., 2018; Chrabaszcz et al., 2018; Smeda et al., 2018).

**Table 1.**
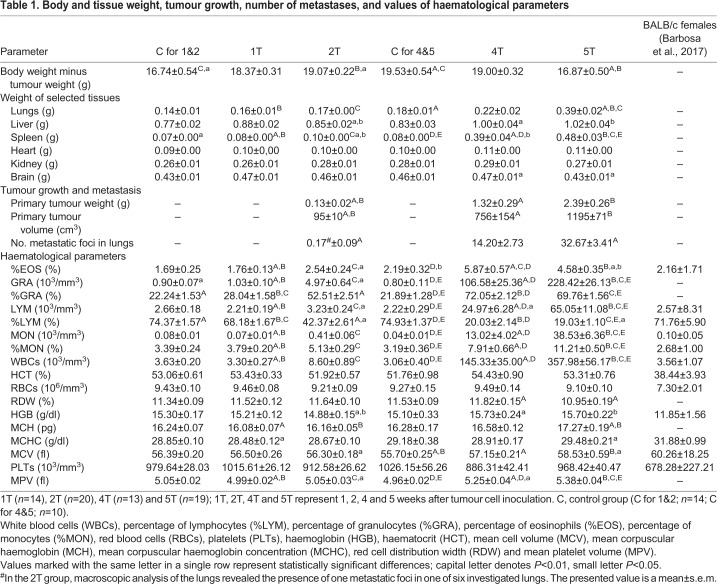
**Body and tissue weight, tumour growth, number of metastases, and values of haematological parameters**

Despite significantly increased weights of primary tumour, lungs, liver and spleen, along with a slight decrease in brain weight, the late metastatic stage was associated with reduced body weight, suggesting cancer-related wasting of the organism. Heart, kidneys and bones from the tumour-bearing mice did not exhibit any abnormal morphological features or any visible tumour foci, and resembled the organs of control animals (Table 1).

### Changes in blood count

The changes in white blood cell (WBC) counts were already visible during the first week after 4T1 tumour cell inoculation but statistically significant differences in blood cell counts were noted in the second week and progressed thereafter. The changes included an increase in total counts of WBCs, granulocytes (GRA), lymphocytes (LYM) and monocytes (MON), and an increase in the percentage of granulocytes (%GRA), monocytes (%MON) and eosinophils (%EOS), with a concomitant reduction in the percentage of lymphocytes (%LYM), although the absolute number of LYM increased (Table 1).

### Changes in plasma concentration of endothelial biomarkers in the early phase of metastasis

**Fig. 2.**
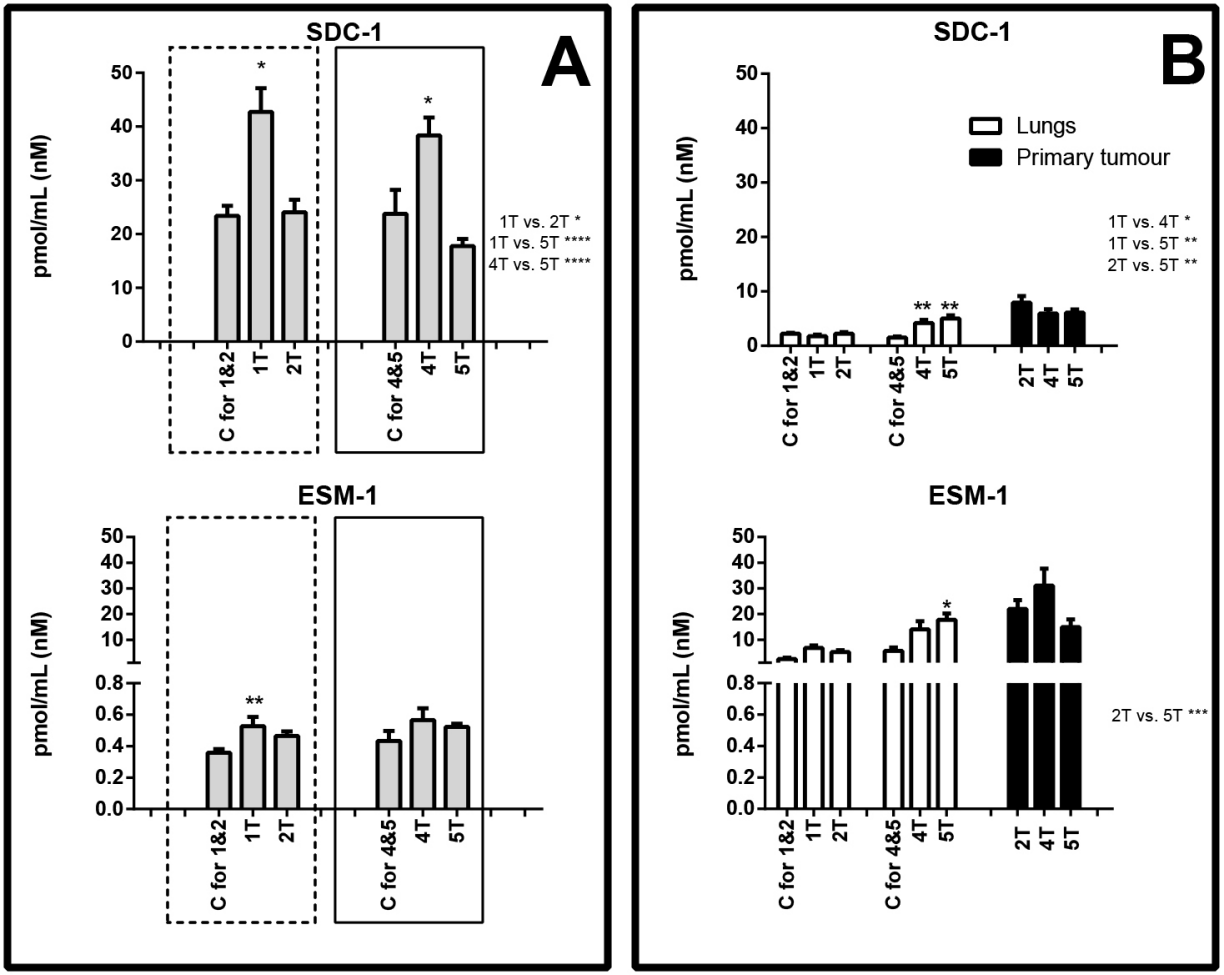
**Concentration of biomarkers of glycocalyx disruption.** Biomarker concentration in plasma (A), and lungs and primary tumours (B) are shown. Areas boxed with a dashed line show proteins that exhibited concentration changes in the early metastatic phase; areas boxed with a solid line show proteins that exhibited concentration changes in the late metastatic phase. C, control group (respective control groups are presented: C for 1&2 and C for 4&5); 1T, 2T, 4T, 5T, groups of tumour-bearing mice in subsequent (1, 2, 4 and 5) weeks after tumour cell inoculation; above the columns statistically significant changes are marked to respective control groups, in primary tumours between the analysed groups; on the right side of the figure are presented statistically significant changes only between the tumour-bearing groups of mice; data are represented as means±s.e.m.; *P<0.05, **P<0.01, ***P<0.001, ****P<0.0001.

**Fig. 3.**
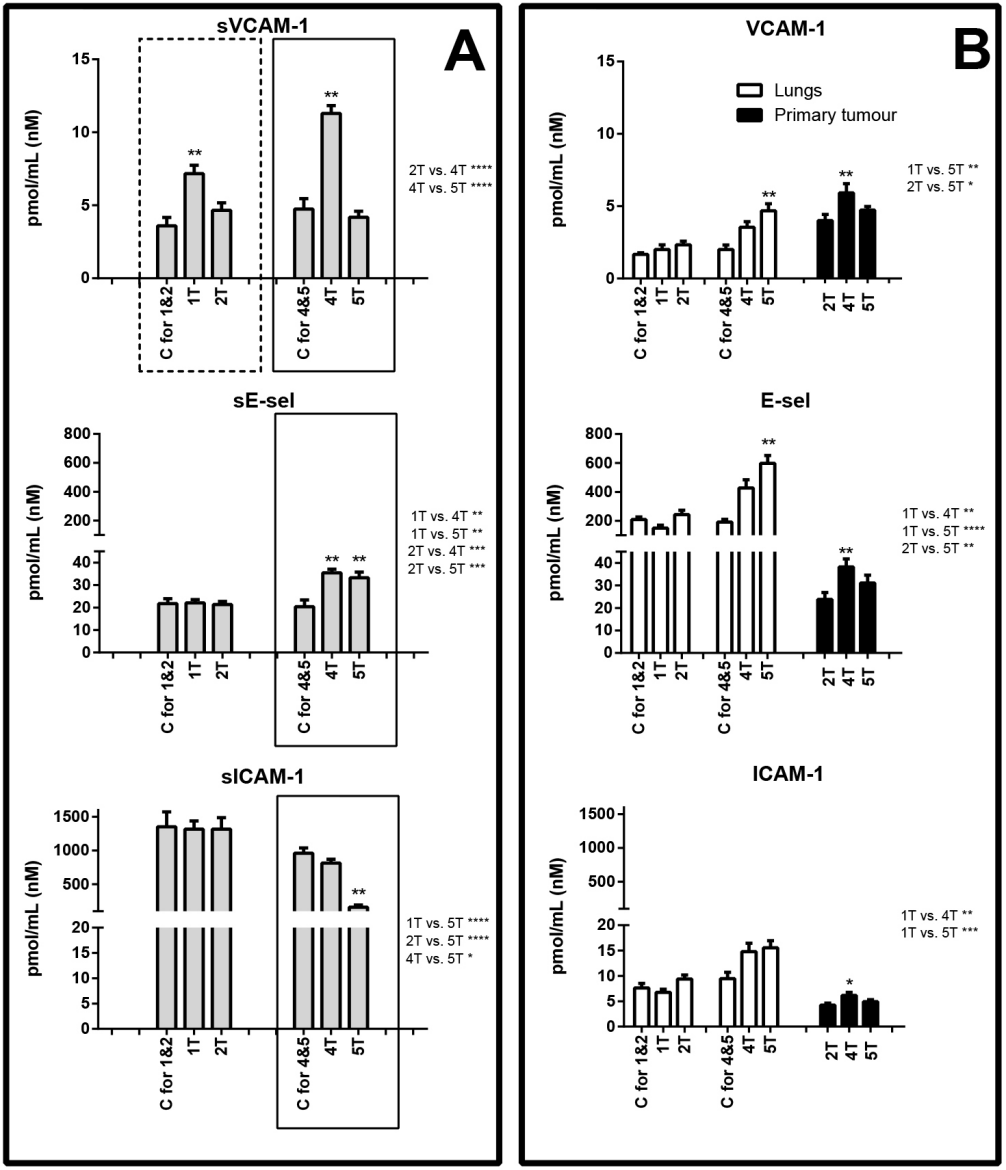
**Concentration of biomarkers of inflammation.** Biomarker concentration in plasma (A), and lungs and primary tumours (B) are shown. Areas boxed with a dashed line show proteins that exhibited concentration changes in the early metastatic phase; areas boxed with a solid line show proteins that exhibited concentration changes in the late metastatic phase. C, control group (respective control groups are presented: C for 1&2 and C for 4&5); 1T, 2T, 4T, 5T, groups of tumour-bearing mice in subsequent (1, 2, 4 and 5) weeks after tumour cell inoculation; above the columns statistically significant changes are marked to respective control groups, in primary tumours between the analysed groups; on the right side of the figure are presented statistically significant changes only between the tumour-bearing groups of mice; data are represented as means±s.e.m.; *P<0.05, **P<0.01, ***P<0.001, ****P<0.0001.

**Fig. 4.**
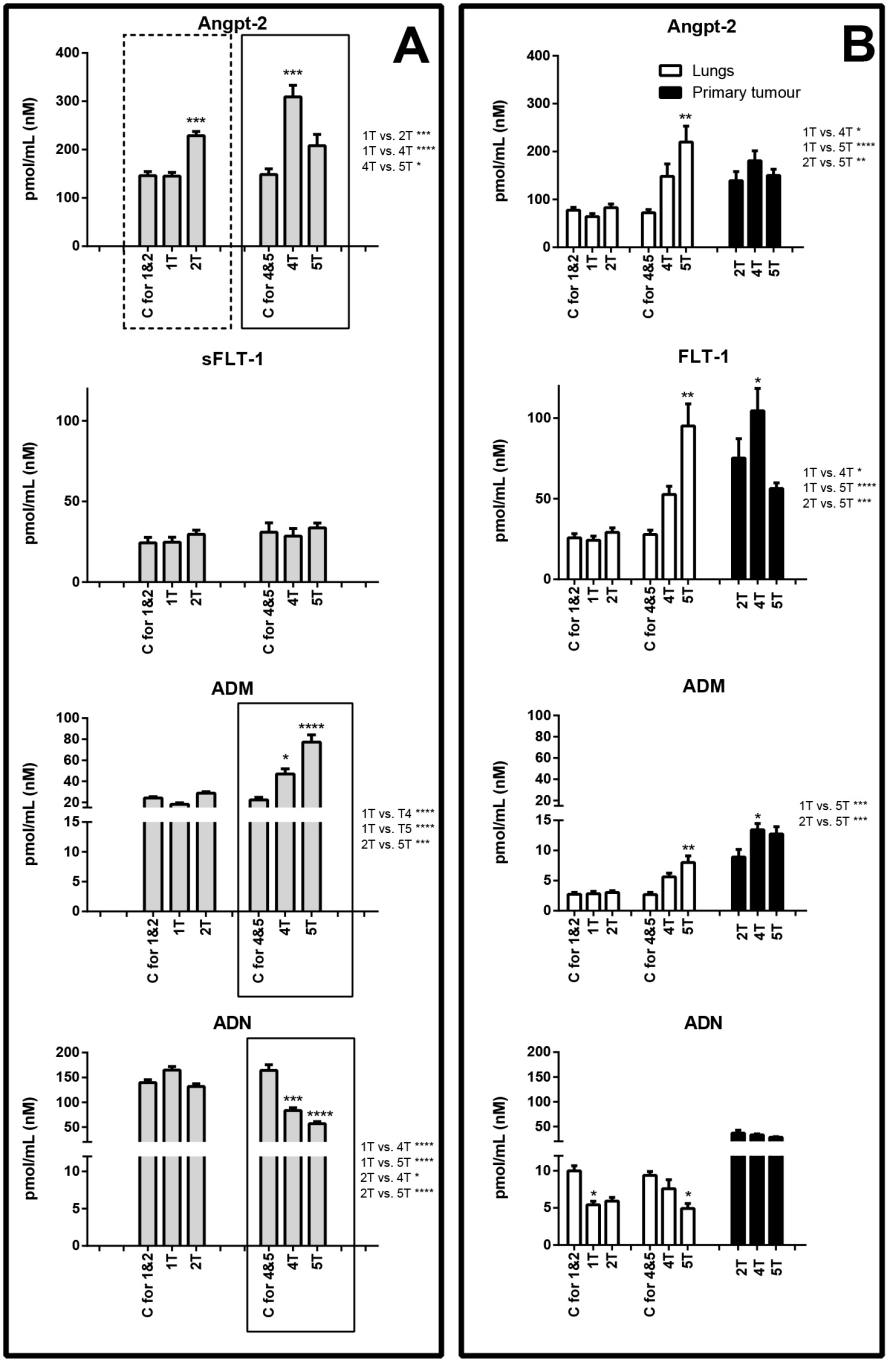
**Concentration of biomarkers of endothelial permeability and biomarkers pathophysiologically linked with breast cancer metastasis (ADM, ADN).** Biomarker concentration in plasma (A), and lungs and primary tumours (B) are shown. Areas boxed with a dashed line show proteins that exhibited concentration changes in the early metastatic phase; areas boxed with a solid line show proteins that exhibited concentration changes in the late metastatic phase. C, control group (respective control groups are presented: C for 1&2 and C for 4&5); 1T, 2T, 4T, 5T, groups of tumour-bearing mice in subsequent (1, 2, 4 and 5) weeks after tumour cell inoculation; above the columns statistically significant changes are marked to respective control groups, in primary tumours between the analysed groups; on the right side of the figure are presented statistically significant changes only between the tumour-bearing groups of mice; data are represented as means±s.e.m.; *P<0.05, **P<0.01, ***P<0.001, ****P<0.0001.

In the first week after cancer cell inoculation, marked glycocalyx disruption (SDC-1 and ESM-1) and notable activation of pro-inflammatory adhesion molecules [soluble VCAM-1 (sVCAM-1)] were detected. Plasma concentration of SDC-1 rose from 23.41±1.88 to 42.71±4.43 pmol/ml and ESM-1 from 0.36±0.02 to 0.53±0.06 pmol/ml (Fig. 2A), whereas sVCAM-1 increased from 3.60±0.57 to 7.17±0.58 pmol/ml (Fig. 3A). In the lungs, during the first week after cancer cell inoculation, only ADN concentration decreased, from 9.98±0.69 to 5.42±0.47 pmol/ml (Fig. 4B).

In the second week after cancer cell inoculation, plasma concentrations of SDC-1, ESM-1 and sVCAM-1 (Figs 2A and 3A) diminished to values observed in control animals, while plasma concentration of Angpt-2 rose from 146.17±8.31 to 228.93±8.57 pmol/ml (Fig. 4A). Plasma concentration of other determined biomarkers did not differ significantly. In the lungs and primary tumours, all biomarkers were measurable, but the differences were not significant.

### Changes in plasma concentration of endothelial biomarkers during the late phase of metastasis

**Fig. 5.**
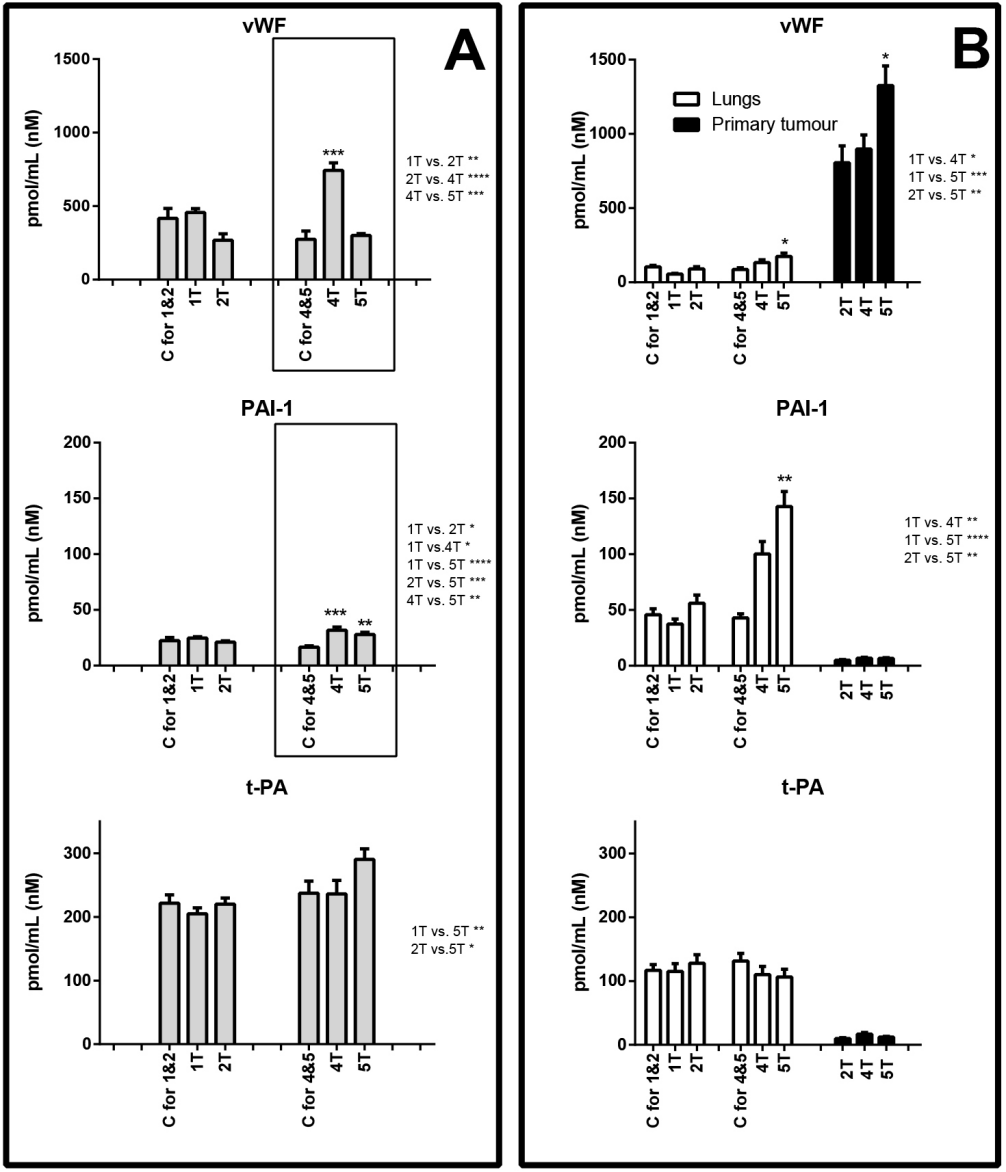
**Concentration of biomarkers of haemostasis.** Biomarker concentration in plasma (A), and lungs and primary tumours (B) are shown. Areas boxed with a dashed line show proteins that exhibited concentration changes in the early metastatic phase; areas boxed with a solid line show proteins that exhibited concentration changes in the late metastatic phase. C, control group (respective control groups are presented: C for 1&2 and C for 4&5); 1T, 2T, 4T, 5T, groups of tumour-bearing mice in subsequent (1, 2, 4 and 5) weeks after tumour cell inoculation; above the columns statistically significant changes are marked to respective control groups, in primary tumours between the analysed groups; on the right side of the figure are presented statistically significant changes only between the tumour-bearing groups of mice; data are represented as means±s.e.m.; *P<0.05, **P<0.01, ***P<0.001, ****P<0.0001.

In the fourth and fifth week after cancer cell inoculation, glycocalyx disruption (SDC-1), endothelial inflammation (sVCAM-1, sE-sel) and increased vascular permeability (Angpt-2) were also noted. However, in contrast to the early phase, a significant rise in ADM plasma concentration and substantial fall in ADN and sICAM-1 concentrations, as well as alterations in the levels of haemostatic biomarkers (vWF, PAI-1), were observed (panel A in Figs 2-5).

In the lungs, during the fifth week after tumour cell inoculation, the concentration of certain proteins increased, e.g. SDC-1 increased from 1.51±0.21 to 5.00±0.60 pmol/ml, VCAM-1 rose from 2.00±0.32 to 4.68±0.50 pmol/ml, E-sel was elevated from 191.53±19.06 to 597.49±54.05 pmol/ml, Angpt-2 increased from 72.12±6.88 to 219.88±33.25 pmol/ml, FLT-1 increased from 27.88±2.62 to 95.20±13.62 pmol/ml and PAI-1 rose from 43.00±3.60 to 142.70±13.62 pmol/ml (panel B in Figs 2-5).

In turn, the primary tumours in the late phase of metastasis exhibited high concentrations of ESM-1 (fourth week: 31.10±6.59 pmol/ml, fifth week: 14.93±2.94 pmol/ml), VCAM-1 (fourth week: 5.92±0.63 pmol/ml, fifth week: 4.73±0.24 pmol/ml), FLT-1 (fourth week: 104.52±13.97 pmol/ml, fifth week: 56.38±3.50 pmol/ml), ADM (fourth week: 13.44±1.04 pmol/ml, fifth week: 12.73±1.21 pmol/ml) as well as vWF (fourth week: 898.23±94.63 pmol/ml, fifth week: 1325.87±132.45 pmol/ml) (panel B in Figs 2-5).

## DISCUSSION

In the present work, a murine model of spontaneously metastasizing 4T1 mammary gland carcinoma with a low number of inoculated tumour cells (1×10^4^) was used, which resulted in a prolonged development of metastases (Buczek et al., 2018; Bailey-Downs et al., 2014). This is in contrast to a number of other studies in which the authors employed 1×10^6^ 4T1 tumour cells (for example, Tao et al., 2008), which led to robust metastases in the lungs, heart, liver, kidney, spleen and bones along with a shorter time period needed to achieve the end-stage of disease. Our approach enabled us to easily discern the early (first-second week) and late (fourth-fifth week) phases of metastasis defined on the basis of metastatic foci formation detected macroscopically in the lungs and by lung histology as also characterised in our previous works (Buczek et al., 2018; Smeda et al., 2018; Chrabaszcz et al., 2018). The early phase of metastasis in this model (first-second week after cancer cell inoculation) was characterised by the presence of the primary tumour (which started to be palpable after the first week following cancer cell inoculation), pulmonary inflammation and micrometastases (Buczek et al., 2018; Smeda et al., 2018). The latter was also confirmed by Fourier-transform infrared (FTIR) spectroscopic imaging, which seemed to be a sensitive approach for the identification and visualisation of early pulmonary micrometastases and macrometastases 2 and 3 weeks after cancer cell inoculation, respectively, in the same murine model of metastatic 4T1 breast cancer (Chrabaszcz et al., 2018; Augustyniak et al., 2018). The late phase of metastasis (fourth-fifth week) was reflected by large primary tumours and numerous metastases easily detected macroscopically as well as robust blood count changes, including pronounced leukocytosis.

The robust increase in WBC count of 4T1 tumour-bearing mice was also reported previously (Wang et al., 2015), and was linked to medullary and extramedullary haematopoiesis (Younos et al., 2011, 2012), with expansion of myeloid-derived suppressor cells (MDSCs) promoting metastases development (Younos et al., 2011; Cao et al., 2014; Kus et al., 2018). The increased leukocytosis underlines robust cancer-associated systemic inflammation as evidenced also by increased plasma concentration of pro-inflammatory cytokines, e.g. interleukin 6 (IL-6), as reported earlier in the same model of 4T1 tumour-bearing mice (Buczek et al., 2018).

The major finding of the present work was the demonstration that, in the first week after cancer cell inoculation, glycocalyx disruption (SDC-1 and ESM-1), endothelial inflammation (sVCAM-1) and increased vascular permeability (Angpt-2) were present, as evidenced by elevated plasma concentrations of respective biomarkers. These results are in line with the early involvement of alterations in the function of pulmonary endothelium in cancer cell extravasation to the lungs (Franses et al., 2013). In fact, the adhesion of cancer cells to the endothelium and subsequent metastasis were shown to be facilitated by endothelial glycocalyx removal (Gasic and Gasic, 1962; Rai et al., 2015). Thus, shedding of endothelial glycocalyx seems to be a prerequisite for the early pulmonary metastasis of 4T1 breast cancer cells. The early phase of metastases formation was also associated with increased plasma concentration of Angpt-2, thereby suggesting changes in endothelial permeability (Felcht et al., 2005). Furthermore, glycocalyx disruption (Cahill and Redmond, 2016; Kurzelewski et al., 2005) was associated with an endothelial NO-deficiency state, relevant to the early pulmonary response to breast cancer metastasis (Tousoulis et al., 2012). Indeed, Smeda et al. (2018) in the same model demonstrated decreased endothelial nitric oxide synthase (eNOS) activity, resulting in a low NO-production state in the pulmonary endothelium during the very early phase of breast cancer pulmonary metastasis, even preceding the onset of its phenotypic switch toward a mesenchymal phenotype (EndMT) (Smeda et al., 2018). Altogether, our results showing increased plasma concentrations of SDC-1, ESM-1, sVCAM-1 and Angpt-2 seem to reflect a local pulmonary endothelial response to murine metastatic breast cancer development involving glycocalyx disruption, endothelial inflammation and increased endothelial permeability that, together with a low-NO pulmonary microenvironment – reported previously in this model (Smeda et al., 2018; Buczek et al., 2018) – might all promote metastases development. These results are fully compatible with the important pathophysiological role of the alterations in endothelial function in metastasis (Mierke, 2008; Gasparics et al., 2016; Wieland et al., 2017).

Given that the pulmonary endothelial surface represents approximately 30% of the total endothelial surface of the cardiovascular system, we claim that an early increase in biomarkers of endothelial dysfunction reflects a pulmonary endothelium response to early metastasis. On the other hand, it seems unlikely that systemic endothelium contributes to these early changes in plasma biomarkers of endothelial dysfunction. We previously demonstrated that the systemic endothelium was not affected in this model until the late stage of metastasis and the occurrence of pronounced systemic inflammation (Buczek et al., 2018).

The late phase of cancer metastasis was also associated with glycocalyx disruption, endothelial inflammation and increased vascular permeability. There were, however, certain differences in the pattern of detected endothelial biomarkers in the late versus early phase of metastasis.

During the late phase of metastasis, concentrations of not only sVCAM-1 but also sE-sel were significantly increased within plasma. Interestingly, the concentration of E-sel in the lungs was also considerably raised, suggesting that pulmonary endothelium could constitute the major cellular origin of sE-sel during the late phase of pulmonary metastasis (Fig. 3), in agreement with a number of reports showing the role of sE-sel in cancer cell progression, for example in homing of metastatic cancer cells (Gogali et al., 2010). In contrast to sVCAM-1, the plasma concentration of sICAM-1 fell considerably during the late phase of metastasis. This finding is surprising, as is the parallel fall in SDC-1, sVCAM-1, Angpt-2 and vWF in plasma in the fifth week after cancer cell inoculation, and therefore requires further study (Zhang et al., 2018).

The important difference in the pattern of plasma biomarkers in early and late phases of metastasis was the late alterations in haemostasis, as evidenced by increased plasma concentration of vWF and PAI-1 without any significant changes in t-PA (Fig. 5A). Interestingly, PAI-1 concentration increased not only in plasma, but also in the lungs, while vWF rose in primary tumours, suggesting a possible different cellular source of these two major regulators of haemostasis: lungs and tumour cells, respectively. Indeed, PAI-1 is known to be produced by pulmonary endothelium (Muth et al., 2004) and its plasma concentration correlated with tumour progression (Lampelj et al., 2015).

In turn, high expression of vWF in primary tumours is compatible with the notion that vWF is required in the initial phase of metastatic foci formation, independent of its role in haemostasis (Mochizuki et al., 2012; Zucker and Cao, 2012). Still, in our hands, a rise in plasma vWF concentration was noted rather late, suggesting that vWF may be implicated during the late phase of murine breast cancer metastasis.

The interesting finding was that the late phase of breast cancer metastasis was associated with a rise in ADM and fall in ADN plasma concentration (Fig. 4A). Hinson et al. (2000) and Oehler et al. (2003) reported that ADM plays an important role in tumour angiogenesis in breast cancer by increasing the intra-tumour blood flow as well as the development of lymph node metastases. In fact, in our hands, we detected an increased concentration of ADM not only in plasma, but also in the primary tumour, suggesting that the growing primary breast tumour might have contributed to the increased plasma concentration of this molecule (Keleg et al., 2007).

A substantial fall of ADN concentration in plasma was associated with a significant fall in ADN content in lungs but not in the primary tumour. Thus, it might be assumed that cancer-wasted adipose tissue, the major cellular origin of a decreased ADN concentration in plasma, was associated with a reduction of ADN within the lungs. The role of ADN in cancer progression is still controversial. It has been shown that ADN inhibits adhesion, invasion and migration of breast cancer cells (Saxena and Sharma, 2010). In the study of Man et al. (2010), adiponectin treatment significantly inhibited liver tumour growth and metastasis by suppression of tumour angiogenesis. By contrast, Landskroner-Eiger et al. (2009) and Denzel et al. (2009) showed that breast cancer tumour vascularisation is stimulated by ADN.

Even though, during the late phase of metastasis, the concentration of FLT-1 increased in the lungs, the plasma concentration of sFLT-1 remained unchanged. Apparently, in the setting of breast cancer metastasis, systemic Angpt-2-dependent mechanisms may be more important than systemic vascular endothelial growth factor (VEGF)-dependent mechanisms, even though the latter is of importance in patients with breast cancer (Thielemann et al., 2010). In patients with gastric cancer, elevated Angpt-2 was strongly associated with liver metastasis (Hacker et al., 2016), suggesting that increased plasma concentration of Angpt-2 may indeed represent an important biomarker of increased endothelial permeability associated with the progression of various types of cancer.

Determination of biomarkers in lung homogenates and primary tumours indicated a possible source of their elevated concentration in plasma. Accordingly, pulmonary endothelium of metastatic lungs seems to contribute to the number of plasma biomarkers raised during the late phase of metastasis (PAI-1, Angpt-2, sFLT-1, sE-sel), whereas primary tumours were an important source of vWF, the concentration of which progressively increased in primary tumours and in plasma along with the progression of breast cancer.

Altogether, even though all selected proteins analysed here were previously reported to be relevant to cancer development [ESM-1 (Yang et al., 2015), PAI-1 (Andreasen, 2007; Duffy et al., 2014), t-PA (Corte et al., 2005), sFLT-1 (Thielemann et al., 2010), vWF (Luo et al., 2012; Zucker and Cao, 2012), sE-sel (Mann and Tanaka, 2012), sVCAM-1 (Silva et al., 2006), sICAM-1 (O'Hanlon et al., 2002; Schröder et al., 2011), SDC-1 (Szatmári et al., 2015), Angpt-2 (Li et al., 2015)], in the present work, we specifically characterised the profile of their changes during the early and late phases of metastasis. Of course, in the current work, we did not provide the experimental results to show the pathophysiological role of a given pathway represented by a measured biomarker, and this is a limitation of this study.

Nevertheless, to the best of our knowledge, our endothelium-oriented approach that aimed to profile endothelial response to breast cancer development and metastasis is unprecedented. Our results provide novel insights into the endothelial response to breast cancer development and metastasis in the well-validated murine model of breast cancer metastasis. We identified glycocalyx disruption, endothelial inflammation and increased endothelial permeability as important events in early pulmonary metastasis in this model, while the late phase of metastasis was additionally featured by alterations in haemostasis and changes in the adrenomedullin and adiponectin pathways. We believe that our methodology may offer a novel tool to profile endothelial phenotype in humans, not only in cancer but also in various diseases associated with endothelial dysfunction, as has been recently shown in our pilot study in humans (Suraj et al., 2019).

## MATERIALS AND METHODS

### Cell culture

Cells from the murine mammary gland tumour cell line 4T1 were purchased from the American Type Culture Collection (ATCC) in 2015 and then kindly donated by Joanna Wietrzyk’s group (Ludwik Hirszfeld Institute of Immunology and Experimental Therapy, Polish Academy of Sciences, Wroclaw, Poland) in the frame of the collaborative STRATEGMED1/233226/11/NCBR/2015 project. The methodology of preparation of cells for the studies in the 4T1 murine model of breast cancer was established by Joanna Wietrzyk’s group. Cells were always used at the second passage after seeding cells from frozen aliquots and were regularly tested for mycoplasma contamination using the MycoAlert Mycoplasma Detection Kit (Lonza Group Ltd, Basel, Switzerland). Cells were maintained in T75 culture flasks as an adherent monolayer in RPMI 1640 GlutaMAX medium (Gibco, Life Technologies, Carlsbad, USA) supplemented with 10% foetal bovine serum (Gibco, Life Technologies), 1 mM sodium pyruvate (Sigma-Aldrich, St Louis, USA) and antibiotic antimycotic solution (100 units/ml penicillin, 100 μg/ml streptomycin and 25 μg/ml amphotericin B; Sigma-Aldrich). Cells were cultured at 37°C in a humidified atmosphere containing 5% CO_2_.

### Animal model

A total of 90 7- to 8-week-old BALB/c female mice were obtained from the Center for Experimental Medicine (Medical University of Bialystok, Poland). Mice were fed a standard laboratory diet, Altromin 1324 Total Pathogen Free for mice with 3188 kcal/kg, including 24% of calories from proteins, 12% of calories from fat and 74% calories from carbohydrates (Altromin, Poznan, Poland), and had free access to water. Tumour cells of murine mammary carcinoma 4T1 (1×10^4^ cells/mouse) suspended in 0.05 ml of Hanks' Balanced Salt Solution (HBSS) (Gibco, Life Technologies) were orthotopically inoculated into the right mammary fat pad. Analyses were conducted in different numbers of animals (*n*) per each group after 1 (*n*=14), 2 (*n*=20), 4 (*n*=13) and 5 (*n*=19) weeks following tumour cell inoculation. The following abbreviations were used for the consecutive weeks after inoculation: 1T, 2T, 4T and 5T, respectively. Healthy BALB/c mice were utilised as two control groups: the control group for the first and second week (C for 1&2; *n*=14) and for the fourth and fifth week (C for 4&5; *n*=10). The number of animals and procedures in each group were specified according to the approval of the Local Ethical Committee for Experiments on Animals at the Jagiellonian University (Krakow, Poland). Experiments were conducted in accordance with the ethical standards, and according to the Declaration of Helsinki and national and international guidelines, with approval by the authors' institutional review board.

### Blood and tissue collection, and haematological analysis

Blood was collected from the right ventricle under anaesthesia using a mixture of ketamine (100 mg/ml) (Vetoquinol, Gorzow Wielkopolski, Poland) and xylazine (10 mg/ml) (Sigma-Aldrich), and a 0.025 ml mixture for 10 g body weight was injected intraperitoneally. Blood samples were collected into syringes containing dipotassium ethylenediaminetetraacetic acid (K_2_EDTA) (1.6 mg/ml) (Aqua-Med, Lodz, Poland). WBCs, %LYM, %GRA, %EOS, %MON, red blood cells (RBCs), platelets (PLTs), haemoglobin (HGB), haematocrit (HCT), mean cell volume (MCV), mean corpuscular haemoglobin (MCH), mean corpuscular haemoglobin concentration (MCHC), red cell distribution width (RDW) and mean platelet volume (MPV) were determined with the aid of an ABC Vet analyser (Horiba, Kyoto, Japan). The blood not destined for haematological analysis was immediately mixed with MS-SAFE protease and phosphatase inhibitor cocktail (PIC) (Sigma-Aldrich) in a ratio of 100:1 and centrifuged at 664 ***g*** for 10 min at 4°C. The resulting plasma was then transferred into Protein LoBind tubes, split into 30 µl aliquots and stored at −80°C until further use.

### Assessment of primary tumours and number of metastases in lungs

In order to remove the blood and ensure reliable analysis of the biomarkers in the tissue, perfusion of the whole body with phosphate-buffered saline (PBS, pH 7.4) was performed immediately subsequently to blood collection. Primary tumours were dissected from the surrounding tissues, measured with a calliper and weighed. Tumour volume (*V*; in mm^3^) was calculated with the following formula (Jiang et al., 2010) (with length and width of the tumour measured in mm):
(1)
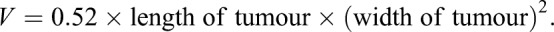


Isolated lungs for biomarker analysis were washed after dissection in sodium chloride 0.9% solution (0.9% NaCl) (VWR, Radnor, USA) and weighed. For histological analysis, lungs were not perfused and were fixed with 4% formalin buffered solution (Chempur, Piekary Slaskie, Poland). The number of metastatic sites in lungs was macroscopically counted. For overall analysis of animal health condition, body weights were checked on the day of tumour cell inoculation and in the weeks thereafter of the experiment. Additionally, the weights of selected organs, namely the lungs, liver, kidney, spleen, brain and heart, were also noted.

### Histopathological analysis

Phosphate-buffered formalin (pH 7.4, 4%) was used for fixing lungs. Haematoxylin and Eosin were used for staining of paraffin-embedded sections (three sections per microscope slide). Ten random images were taken at 200×, and additionally at 400×, with an Olympus light microscope BX51 (Olympus Corporation, Tokyo, Japan).

### Preparation of lung and primary tumour homogenates for biomarker determination

Tissue homogenates of lungs and primary tumours were prepared in PBS with the addition of PIC in a ratio of 100:1 using a Precellys Evolution Tissue Homogenizer (Montigny-le-Bretonneux, France) (chilled with liquid nitrogen, 7500 rpm, 3 cycles, 30 s break). The homogenates were centrifuged (4°C, 10,000 ***g***, 15 min) and the supernatant was frozen at −80°C until analysis.

### MicroLC/MS-MRM analysis of biomarkers

The key part of the study was to establish the concentration of 12 selected biomarkers in mouse plasma, lung and primary tumour homogenates using the microLC/MS-MRM method. For each biological sample from three matrices, the multiplexed analysis of the selected 11 proteins and one peptide was performed in one, single analytical run. The panel included endothelial proteins, specifically SDC-1, ESM-1, VCAM-1, E-sel, ICAM-1, Angpt-2, FLT-1, vWF, t-PA and PAI-1, as well as biomarkers pathophysiologically linked with breast cancer metastasis, including ADN, the protein produced by adipocytes that is known to display vasoprotective and pro-angiogenic activity (Miłosz et al., 2007; Landskroner-Eiger et al., 2009), as well as ADM, a peptide reported to contribute to breast cancer progression also displaying pro-angiogenic activity (Oehler et al., 2003).

The detailed description of the sample preparation for microLC/MS-MRM analysis considering the validation of the analytical method developed for the analytes was included in Suraj et al. (2018, 2019). In brief, total protein concentration in plasma, tissues and primary tumour was assessed using a NanoDrop 8000 spectrophotometer (Thermo Fisher Scientific, Waltham, USA). The samples were diluted to a concentration of 7 mg/ml using 25 mM ammonium bicarbonate (NH_4_HCO_3_) (Sigma-Aldrich) and 210 µg of total protein was denatured with 10% solution of sodium deoxycholate (Sigma-Aldrich) in 25 mM NH_4_HCO_3_, then diluted with 25 mM NH_4_HCO_3_. Samples were reduced with 50 mM tris(2-carboxyethyl)phosphine (TCEP) (Sigma-Aldrich) in 25 mM NH_4_HCO_3_ for 30 min at 60°C and alkylated in the dark with 100 mM iodoacetamide (IAM) (Sigma-Aldrich) in 25 mM NH_4_HCO_3_ for 30 min at 37°C. Next, the excess of IAM was quenched by the addition of 100 mM DL-dithiothreitol (DTT) (Sigma-Aldrich) in 25 mM NH_4_HCO_3_. The incubation of the samples lasted 30 min at 37°C. Digestion was carried out for 16 h at 37°C with sequencing grade modified trypsin (SGM trypsin) in a 50:1 ratio (substrate:enzyme) (Promega, Madison, USA). Prepared internal standard peptide solutions obtained from dissolved powders of internal standard peptides, synthesised and quality controlled by Innovagen (Lund, Sweden) (Suraj et al., 2018, 2019), specific for each target peptide sequence were implemented just before ceasing the digestion. Finally, this process was stopped by the addition of formic acid (FA) (Sigma-Aldrich) at a final concentration of 0.5% v/v. Next, sodium deoxycholate was pelleted. The sample supernatant obtained after centrifugation (3000 ***g*** for 10 min at 23°C) was desalted and concentrated by the micro-solid phase extraction (µSPE) procedure using the Oasis HLB elution plate with 2 mg of sorbent mass per well (Waters, Milford, USA). Briefly, the resin was rinsed with methanol and equilibrated with water (H_2_O). The sample was loaded, washed with water and eluted with a 50% solution of acetonitrile (ACN) (Sigma-Aldrich) in deionized water with an addition of 0.1% FA. Samples were lyophilized and resuspended in 50 µl 20% ACN in H_2_O.

The analysis of all samples was performed using the microLC/MS-MRM method. In the analysed samples, the digested peptide fragments specific for the selected proteins were first resolved using the microLC system (Nexera Shimadzu, Kyoto, Japan). Each sample was loaded (2 µl) to the analytical column: ACE, C8 analytical column (150×1.0 mm i.d., 5 µm, 300 Å, Advanced Chromatography Technologies Ltd, Aberdeen, UK). The mobile phases consisted of 0.1% FA in ACN v/v and 0.1% FA in H_2_O (v/v). The total time of analysis was 50 min in a gradient elution. The flow rate was 100 µl/min.

For mass detection, a highly sensitive mass spectrometer QTrap 5500 (Sciex, Framingham, USA) was applied. The mass spectrometer was operated with the following parameters: ion spray voltage: 5500 V; source temperature: 400°C; curtain gas: 25 psi; ion source gas 1: 35 psi; ion source gas 2: 50 psi. The values for collision energy (CE), declustering potential (DP), entrance potential (EP) and collision cell exit potential (CXP) were specific for each transition. Two transitions free of signal interferences when present in a plasma-digest background were selected as the final ion pairs for use in the final assay. A list of transitions and optimisation parameters were presented in detail in methodological publications by Suraj et al. (2018, 2019).

The obtained data were processed by Analyst software version 1.6.2 developed by the SCIEX company (Framingham, USA). All integrated peaks were checked to ensure correct peak detection.

### Statistical analysis

Data were presented as means±s.e.m. Assessments of normality (Shapiro–Wilk test) and equality of variances (Levene test) were performed. To assess the statistical significance of the results, a one-way analysis of variance (ANOVA) with Tukey's *post hoc* test or a non-parametric Kruskal–Wallis test were performed (two-tailed). The results were evaluated with Statistica 12.0 software (StatSoft, Tulsa, USA), with *P*-values of less than 0.05 regarded as statistically significant.

Results were presented in comparison to the two control groups. Despite alterations between these control groups, there were no statistically significant changes between them except for the animal weights, which differed significantly.

**Fig. 6.**
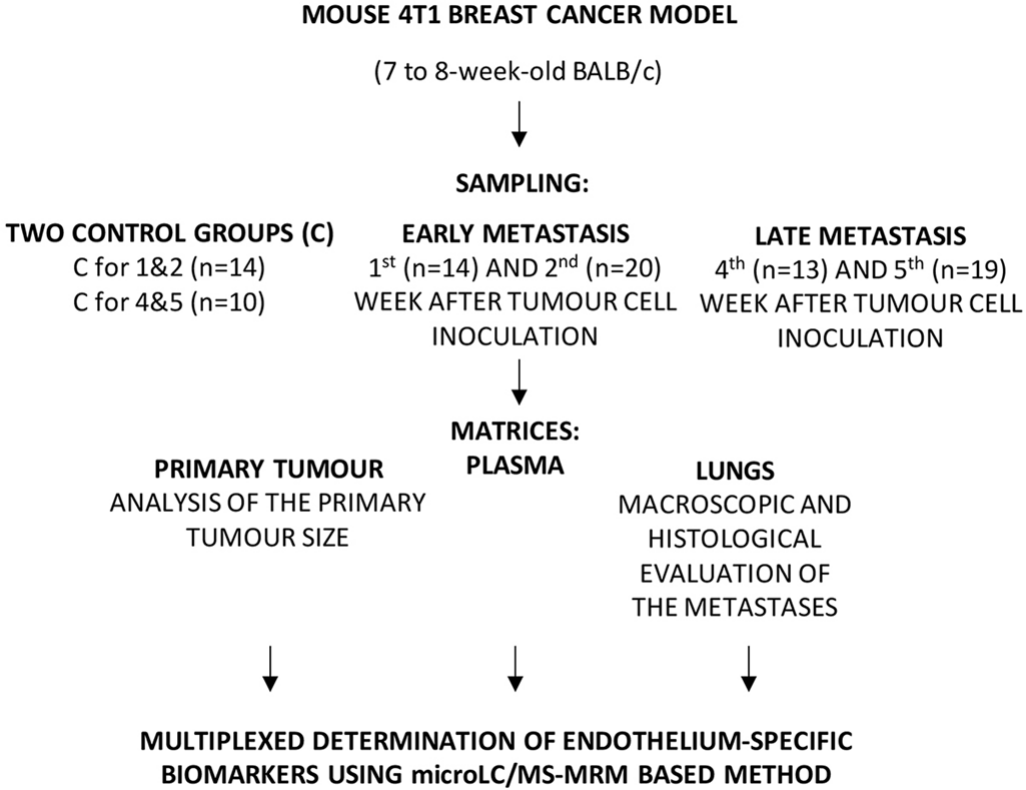
**Summary workflow diagram of the study design.**

A summary workflow diagram of the study design is portrayed in Fig. 6.
